# A Financial and Global Demand Analysis to Inform Decisions for Funding and Clinical Development of Group B *Streptococcus* Vaccines for Pregnant Women

**DOI:** 10.1093/cid/ciab782

**Published:** 2021-11-02

**Authors:** Stefano Malvolti, Clint Pecenka, Carsten F Mantel, Melissa Malhame, Philipp Lambach

**Affiliations:** 1 MMGH Consulting, Zurich, Switzerland; 2 PATH, Seattle, Washington, USA; 3 Department of Immunization, Vaccines and Biologicals, World Health Organization, Geneva, Switzerland

**Keywords:** group B *Streptococcus*, neonatal sepsis, demand forecast, financial evaluation, vaccine

## Abstract

**Background:**

Despite group B Streptococcus (GBS) being a leading cause of maternal and infant morbidity and mortality, no vaccine is currently available. To inform vaccine developers, countries, and funders, we analyzed the key factors likely to influence the demand for a GBS vaccine and the long-term financial sustainability for a vaccine developer.

**Methods:**

Using population-based forecasting, we estimated the demand for a GBS vaccine; using a discounted cash flow model we estimated the financial viability for a vaccine developer.

**Results:**

Demand for this vaccine can be significant if countries adopt policy recommendations for use, in particular, the largest ones, most of which have a burden that justifies use of the vaccine, and if financing for the vaccine is made available either by countries or by funding mechanisms such as Gavi, the Vaccine Alliance.

**Conclusions:**

This analysis suggests the potential for financial and commercial viability for a vaccine developer pursuing the commercialization of a GBS vaccine. Risks exists in relation to the clinical trial design and costs, the level of competition, countries’ ability to pay, the administration schedule, and the availability of policies that encourage use of the vaccine. To reduce those risks and ensure equitable access to a GBS vaccine, the role of donors or financers can prove very important, as can a coordinated operational research agenda that aims at clarifying those areas of uncertainty.

**KEY FINDINGS**

**1. Data Inputs**

Based on population projections, vaccination and maternal services coverage data, maternal immunization systems strength estimates, and other standard forecasting and financial parameters, we developed a demand forecast for a group B *Streptococcus* (GBS) vaccine and performed a discounted cash flow analysis for a commercial entity developing such a vaccine.

**2. What Is New?**

In this first attempt to quantify the global demand for a GBS vaccine and to estimate the financial viability for a vaccine developer under different scenarios, we concluded that, albeit with some risk areas, the development effort should be potentially sustainable and attractive for a commercial entity.

**3. What To Do?**

Actions are required to ensure availability of policies at national and global levels that encourage use of the vaccine in high- and low-resource settings, as well as engagement of donors or financers that, by de-risking the development of the GBS vaccine, can speed up the progression toward marketing authorization.

**4. Key Gaps**

A coordinated operational research agenda will be essential for clarifying specific areas of uncertainty that are likely to influence the developers’ decisions, particularly countries’ interest for a GBS vaccine and their willingness and ability to pay.

GBS is a leading cause of sepsis and meningitis in neonates and young infants and an important cause of stillbirth. Globally, GBS disease is estimated to cause at least 409 000 (UR, 144 000–573 000) cases of serious infections in mothers, their fetuses, or infants and to cause147 000 (Uncertainty Range [UR], 47 000–273 000) stillbirths and infant deaths annually [1]. Despite being home to only 13% of the world’s population, Africa has the highest burden, with 54% of estimated cases and 65% of stillbirths and infant deaths [2]. Because of the significant burden of disease and the technical feasibility, the World Health Organization (WHO) Product Development for Vaccines Advisory Committee identified GBS maternal vaccination as a priority. Priority activities have been highlighted by WHO for vaccine researchers, funders, and product developers, with the goal of accelerating the pathway to vaccine availability [3–5].

Several GBS vaccines are in development by large multinational and smaller companies, partly supported by donor funding [6]. Several programs have failed or have been discontinued, and none of the vaccines currently in clinical development have progressed beyond phase 2 [7]. The largest investment decisions prior to realization of a licensed vaccine are therefore still to be taken.

Those decisions are primarily influenced by the size of the target population, the revenue potential, the required investment, the clinical development feasibility, and the regulatory feasibility [8]. To fill the gap for the first 3 factors mentioned above, we developed high-level estimates for the potential global demand and revenues for a GBS vaccine that is aligned with WHO-preferred product characteristics and with policy recommendations from global policy-making bodies such as the WHO Strategic Advisory Group of Experts on Immunization. In addition, we assessed the potential need for nonmarket financial incentives by estimating the return on investment and financial sustainability of such a vaccine.

## OBJECTIVES

This article is the ciab795, ciab796, ciab785, ciab768, ciab770, ciab777, ciab767, ciab769, ciab776, ciab784 series of articles from this GBS series. There are 2 objectives of this article: to provide a first estimate of the global demand for a GBS vaccine across countries of all income levels and to assess the financial viability for a vaccine manufacturer to develop and commercialize a GBS vaccine

## METHODS

A global strategic demand forecast was built to perform the analyses in 182 countries across all income levels. (Of the 194 WHO member states the 12 smallest countries were not included in the analysis, those countries representing less than 1% of the global population in analysis.) Those countries are forecasted to represent 136 million live births in 2030 [9]. A discounted cash flow (DCF) model was built to assess the financial viability from a vaccine developer standpoint. The country economic perspective is covered in a different article in this GBS series.

### Demand Forecast 

**Figure 1.**
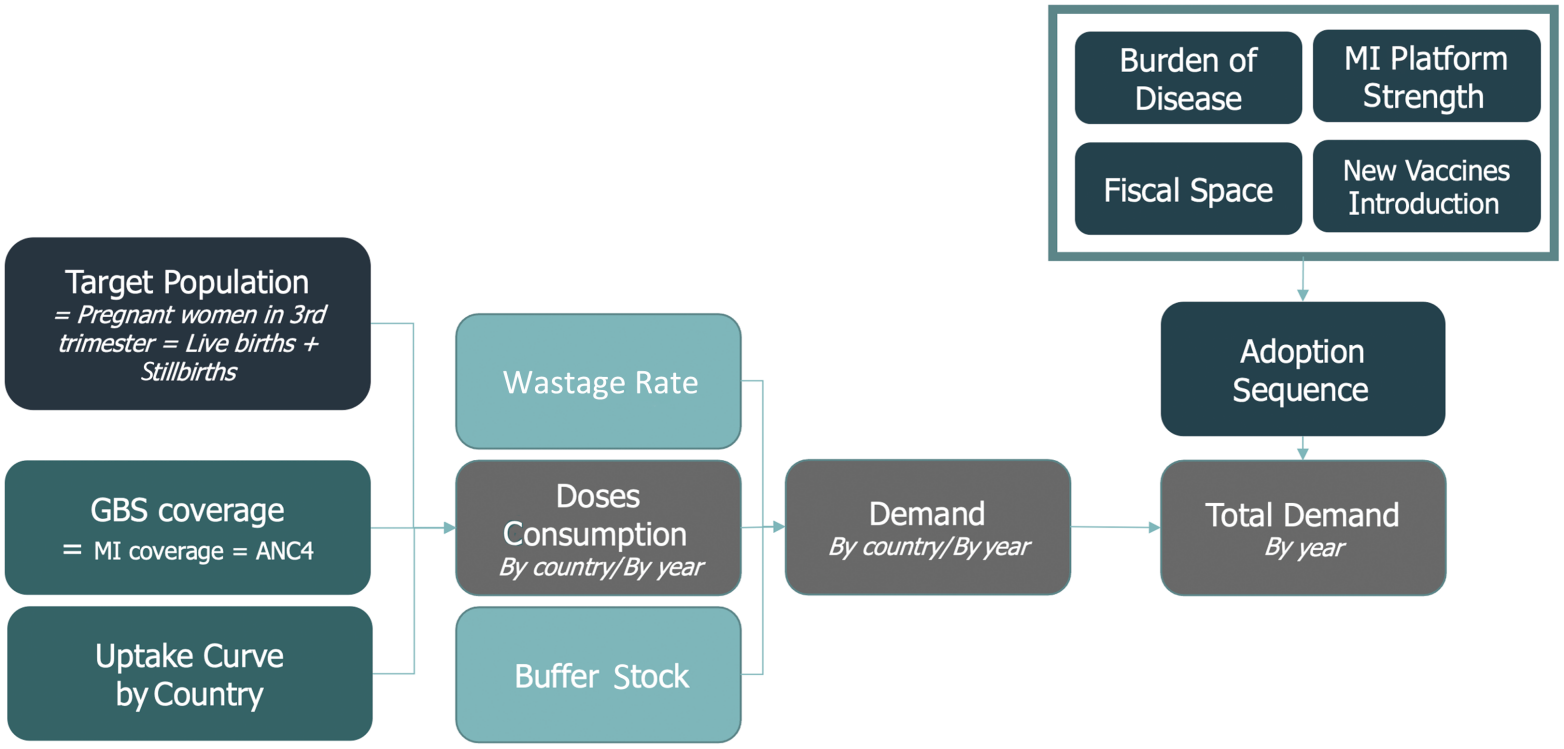
Flow diagram of the demand forecasting process. Abbreviations: ANC, antenatal care; GBS, group B *Streptococcus*; MI, maternal immunization.

The demand forecast was developed using a standard population-based forecasting approach [10–12], as illustrated in Figure 1, estimating the potential demand for a GBS vaccine in each country. The estimate was based on the size of the target population in the different scenarios, the attainable coverage, and the specific uptake curve of the vaccine, estimating the global adoption sequence based on the following 4 country-specific factors: disease burden, the strength of the maternal immunization platform, the available fiscal space, and the track record in earlier introduction of new vaccines.

### Financial Analysis

For the financial analysis, a standard DCF methodology was used to calculate the project’s net present value (NPV). Standard DCF evaluations [13] discount the financial flows generated by a single project by using the weighted average cost of capital (WACC) that captures the specific risk of the business in analysis via the estimate of the cost of equity for that type of business per the capital asset pricing model. The expectation of additional returns in reward for projects that can be considered riskier than the average portfolio is captured by increasing the discount rate beyond the WACC in what is defined as a “hurdle rate.” A hurdle rate was defined in this evaluation.

To calculate the project’s NPV, a simplified profit and loss statement was built composed of the following items: *revenues:* for each year, based on the doses required by the program as calculated in the demand forecast, the total revenues were determined based on the formula, doses deployed × price per dose; *cost of goods sold (COGS):* for each year, based on the doses required by the program as calculated in the demand forecast, the total costs of producing the doses of vaccine sold were computed based on the formula, doses deployed × COGS per dose; *costs of clinical development:* for each year, the total cash disbursement required to finance the clinical development program, inclusive of cost of clinical trials, cost of clinical trial material, and cost of regulatory processes, was calculated by adding all components. Those costs are expensed in the year when they incur and therefore represent cash outflow; and *investment in the manufacturing facility:* for each year, the cash disbursement for the construction of the manufacturing plant was estimated. Those costs were capitalized and depreciated. Their disbursement contributes to the calculation of the free cash flow.

Depreciation; sales, general and administrative (SG&A) expenses; and taxes were also factored in to determine the net profit. Those parameters provided the basis for the calculation of the free cash flow and of the NPV according to the standard DCF method, applying the appropriate discount rate for the relevant industry per the following formula:


$$\begin{array}{l} {\Sigma_{t=0}}^{n}Free\;Cash\;Flow^{t}/\;{\left(1\;+\;Discount\;Rate\right)}^{t} \end{array}$$

where t = number of time periods (ie, years).

No residual value for the cash flow streams generated after 2040 was included in this analysis.

### Scenarios

For each parameter in the demand forecast and in the DCF analysis, a set of assumptions was defined based on the best available information. Scenarios were constructed to understand the impact of different designs of the vaccination program (single dose, 2 doses in pregnancy, and prime dose in adolescence followed by booster dose in pregnancy) as well as to assess the impact of key variables (number of vaccine competitors, the type of clinical trials, COGS level, and existence of Gavi, the Vaccine Alliance [Gavi] financing).

### Demand Forecasting Assumptions

For each variable in the model, the WHO GBS Scientific Advisory Group defined and validated assumptions in meetings and teleconferences that occurred in late 2019 and 2020.

#### Vaccine Characteristics

The characteristics of the GBS vaccine used for the base forecast have been aligned with the preferred product characteristics (PPC) published by WHO [14]. Specifically, the assumption of a single-dose schedule has been adopted; this is reflective of the candidates that have progressed further in clinical development [7]. The characteristics most relevant for this work are captured in Table 1.

**Table 1.** Selected Parameters from the World Health Organization Preferred Product Characteristics

Parameter	Assumed Characteristic
Indication	Prevention of laboratory-confirmed GBS stillbirth and invasive GBS disease in neonates and young infants
Target population	Pregnant women in the second or third trimester of pregnancy
Schedule	1 dose
Safety	At least as favorable as influenza, tetanus toxoid, and acellular pertussis vaccines
Efficacy	80% protection against GBS-conformed stillbirth and invasive disease in offspring
Serotype coverage	>90% of current invasive disease isolates in the target region
Route of administration	Intramuscular
Presentation	Single-dose packaging
Year of first global registration	2028
Prequalification year	2029 (first country introduction in 2029)
World Health Organization recommendation	Recommended for all pregnant women in countries with substantial disease burden
Value proposition	Similar economic barriers as other new EPI vaccines

Abbreviation: EPI, Expanded Program on Immunization; GBS, group B *Streptococcus*; IM, Intramuscular.

As part of the scenario analysis, the following vaccine characteristics that differ from those established in the PPC have been considered: a 2-dose schedule administered during each pregnancy and a 2-dose schedule with a first dose given concurrently with the human papilloma virus (HPV) vaccine in adolescence and the second dose during each pregnancy.

#### Target Population

Women between 24 and 34 weeks of pregnancy were assumed as the primary target population for the 2 doses delivered through antenatal care (ANC) services during 2 of the 4 scheduled pregnancy visits. Pregnant women were assumed as being vaccinated during each pregnancy. In the absence of reliable data on the number of pregnant women per country, the size of this population was derived by adding live births and stillbirths. Estimates of live births for the period 2026–2040 were sourced from the United Nation the database of the Population Division of the United Nations’ Department of Economic and Social Affairs (DESA’s) Population Division database [9]. The stillbirth data from 2015 [15] were extrapolated by applying the average rate of change from 2000 to 2015 to estimate the values for 2026 and beyond. The addition of 2 million stillbirths in 2030 represented 1% of pregnant women globally and 2% of pregnant women in low-income countries (LICs). The negative impact of multiple births on the calculation of the number of pregnant women was assessed and found to be of comparable impact (2%–3%) [16]. However, at the time of forecasting, no consistent source for country data was found and thus no adjustment for multiple births has been included in the forecast.

The scenario that includes delivery of the first dose of the vaccine at the same time as the HPV vaccine assumed 10-year-old girls as the target group across countries. Population data through 2035 [9] were available from the dataset supporting the WHO MI4A (Market Information for Access to Vaccines) HPV forecast [17] and were extended using the linear forecasting function of Excel.

#### Coverage

Different methods were used to derive coverage estimates for the different scenarios mentioned above.

##### Estimate of the Coverage of the First (or Single) Dose Delivered During ANC. 

Coverage for 110 low- and middle-income countries (LMICs) was retrieved from the model of the gestational age timing and coverage of each of the 4 ANC visits per country developed to estimate coverage in a forecast for a respiratory syncytial virus (RSV) vaccine delivered through maternal immunization [18]. Those estimates were compared to unadjusted ANC1, ANC4, and protection-at-birth values. It was found that the unadjusted ANC4 values [19] resulted in a good approximation of the adjusted values used for RSV. Based on this, ANC4 was used for the 74 higher-income countries not included in the RSV forecast.

To generate estimates for the period 2027 to 2040, RSV coverage estimates from 2026 were increased 1 percentage point per year for those countries with RSV coverage below 95% until it reached 95% at which point it was held stable throughout the remaining forecast period. If a country RSV coverage rate was already above 95% in 2026, it remained stable throughout the forecast period.

ANC visit data are available from 1993–2018 [19], with more than 50% of data from 2014 or more recent. To extend the last reported ANC4 data to the period 2026–2040, a standard increase of 1 percentage point per year was applied to all countries with rates below 90% until the rate reached 90% at which point it was kept stable throughout the forecast period. If the rate was already above 90%, it was kept stable throughout the forecast period. This more conservative approach, compared to the RSV coverage, was adopted to reflect the high level of uncertainty linked to the GBS vaccine development. A list of countries is provided in the Supplementary Materials with the coverage rates used.

##### Estimate of the Coverage of the Second Dose Delivered during ANC. 

Given that for all vaccines some people who receive the first dose do not receive the second (drop-out effect), a factor of 0.9 was applied to the first/single-dose coverage to estimate the coverage level of the second dose delivered during ANC. In the absence of solid data sources, this approach was validated by the WHO GBS Scientific Advisory Group.

##### Estimates of the Coverage for the Priming Dose Delivered in Adolescence. 

Coverage of the HPV vaccine delivered in adolescence was retrieved from the global demand forecast developed in 2019 by the WHO MI4A initiative. This forecast included a scenario in which 131 countries would use a 1-dose HPV vaccination regimen starting in 2022 [17]. The forecast assumed the target population to be girls aged between 9 and 14 years, which is in line with the WHO recommendation. The estimated coverage for the single dose of HPV vaccine was used in the GBS forecast for the years 2026–2030. For the remaining 51 countries that were assumed to continue using a 2-dose HPV regimen in the HPV forecast, the blended coverage of both first and second dose was used as the basis for this GBS forecast. In addition, this blended coverage was adjusted upward by a factor of 1.1 and capped at 99% to account for the higher likelihood of 1 dose being administered vs 2. Coverage data from 2026 to 2030 were extended through 2040 using the linear forecast function of Excel.

#### Adoption Sequence

This forecast was developed 9 years before the first expected vaccine marketing authorization; such a time gap prevents having solid insights into an individual country’s year of introduction of a GBS vaccine in their immunization schedule. Therefore, a predictive algorithm was designed to define a logical adoption sequence based on 4 factors representing the potential local importance of GBS vaccination and an individual country’s financial and programmatic readiness to implement a vaccination program.

##### Burden of Disease. 

Countries were assigned a value based on the rate of early-onset deaths caused by GBS disease [1] per live birth stratified by their sustainable development goal subregions. (Southern Asia, Eastern Asia, Central Asia, West Asia, SE Asia, Northern Africa, Southern Africa, Western Africa, Mid. Africa, Oceania, Caribbean, Central America, Developed.) Early-onset deaths represent two-thirds of the GBS deaths [1]; henceforth, we assumed this metric to be the one where awareness is higher among policy makers when discussing country adoption decisions. Where country-specific data were available, they were used to determine whether they would change the country categorization; however, in each case, the country ranking remained unchanged both individually and by subregion. Countries were ranked from highest to lowest early-onset mortality and assigned a score of 3 for highest burden and 1 for lowest based on thresholds drawn at notable and evenly spaced natural changes in the slope of the curve for all countries. Within regions with a rate of <0.0005 per million births, countries were assigned a score of 1; countries within regions with a rate between 0.0005 and 0.00122 per million births were assigned a score of 2; and countries within regions with a rate above 0.00129 were assigned a score of 3.

##### Fiscal Space. 

Countries were ranked according to the percentage of general government expenditure spent on health. This indicator was chosen with reference to the Abuja Declaration that set a goal for health spending of 15% of government revenue for countries. While this declaration is focused on the African region, this target is also widely referred to in other regions [20]. The health expenditure as percentage of government revenue indicator was available for 97% of the countries in scope [21]. A score of 1 was assigned to countries spending <7.5% on health, a score of 2 was assigned to countries spending more than 7.5% but less than 15%, and a score of 3 was assigned if their spending was ≥15%.

##### Maternal Immunization Platform Strength. 

Countries were ranked based on the strengths of their maternal immunization platform as measured by the maternal immunization and antenatal care situation analysis (MIACSA) [22] project in terms of their potential to protect mothers and young infants from vaccine-preventable diseases. Countries classified by the MIACSA project in groups 1 and 2 were considered to have low functionality and were assigned a score of 1, countries in group 3 were assigned a score of 2, and countries in group 4 were assigned a score of 3. Three high-income countries (HICs) not included in the MIACSA analysis were assigned a score of 3.

##### Vaccine Introduction History. 

A composite indicator of country introduction status as of July 2019 for pneumococcal conjugate vaccine (PCV), rotavirus vaccine (RVV), and HPV vaccine was created. While none of these vaccines are indicated during pregnancy, the 3 vaccines were selected because of their “global” scope (eg, meant to be introduced in all countries), “recent” rollout (compared with diphtheria tetanus pertussis–containing vaccines that were introduced decades ago), and “routine” delivery approach. If a country had introduced none of the 3, a score of 0 was assigned; a score of 1 was assigned if 1 of the vaccines was introduced; a score of 2 if 2 vaccines were introduced; and a score of 3 for 3 introduced vaccines. If a country had introduced HPV vaccine at a subnational level or into a pilot program, it was counted as having not introduced.

Each country was attributed a total unweighted score resulting from the simple addition of the scores from each of the 4 indicators. Based on their total unweighted score, ranging between 3 and 12, countries were assigned to different clusters.

**Figure 2.**
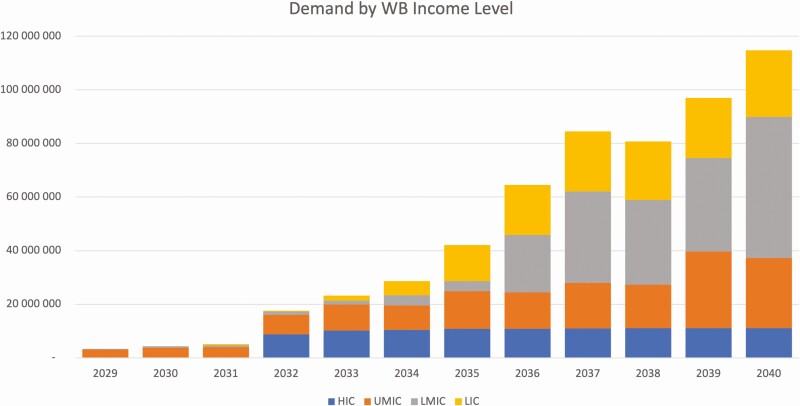
Vaccine demand in doses stratified by income level. Abbreviations: HIC, high-income country; LIC, low-income country; LMIC, lower middle–income country; UMIC, upper middle–income country; WB, World Bank.

As a result of this scoring mechanism (Figure 2), 20 countries with scores of 11 and 12 were assigned to the “early adopters” cluster assumed to introduce in years 1 to 3 from first registration. Sixty-seven countries with scores of 9 and 10 were assigned to the “followers” cluster assumed to introduce in years 4 to 6 after first registration, and 78 countries with scores of 6, 7, and 8 were assigned to the “late adopters” cluster assumed to introduce in years 7 to 9 after first registration. Among those, 9 countries eligible for Gavi support per 2020 that scored 5 and were moved “up” from the “nonadopter” cluster to capture the influence that the financial contribution from Gavi could have on addressing domestic financial constraints that could limit adoption of vaccines otherwise considered priority by those countries. Finally, 16 countries with scores of 5 and below were assumed not to introduce during the forecast period in the baseline forecast and assigned to the nonadopters cluster. See the Supplementary Materials for a list of countries in each category and their total unweighted scores.

An annual introduction sequence was then derived from the country groupings whereby the total birth cohort of all countries in each group was divided into thirds and countries were assigned to introduce in descending order based on their scores starting in the first year of their cluster period until one-third of the birth cohort was reached. After that, countries were assigned to the second year of their cluster period up to exhaustion of the second third of the birth cohort. Finally, the remaining countries were assigned to the third year of their cluster period. The assignment of countries evenly throughout a decade was considered the best way of approximate patterns of introduction observed globally with other new vaccines, while being mindful that the exact timing of a specific country introduction is one of the least certain elements of any forecast.

#### Other Demand Forecasting Parameters

##### Uptake Curve. 

Following standard global vaccine forecasting conventions [23], for most countries the full target population, that is, pregnant women estimated as indicated above or a mix of girls aged 9–14 years and pregnant women in the prime-boost scenario, was assumed to be eligible in year 1 of vaccine introduction. For large countries with more than 10 million pregnant women, 80% of the target population were assumed to be eligible for vaccination in year 1 and 100% of this population in year 2.

##### Wastage Rate. 

For vaccines in single-dose packaging, the standard 5% wastage rate was applied [24].

##### Buffer Stock. 

Twenty-five percent additional vaccine stock was assumed to be needed in year 1 to top-up the supply channel. In addition, 25% of the incremental demand in subsequent years was also included.

### Financial Assumptions

#### Vaccine Prices

Vaccine price estimates were defined by benchmarking prices of some of the most adopted vaccines among the ones developed in the last 20 years. Price benchmarking of PCV, RVV, and HPV for a range of country income levels was conducted, averaging the 2019 country values from the WHO MI4A vaccine price database (Table 2).

**Table 2.** Pneumococcal Conjugate Vaccine, Rotavirus Vaccine, and Human Papilloma Virus Average Price Per Dose Stratified by Country Income Group

Country income level, 2018 sample size	Average Price per Dose, US Dollars					
	2013	2014	2015	2016	2017	2018
High-income country, n = 49	41	45	36	47	45	49
Upper middle–income country, n = 34	51	21	15	16	20	17
Lower middle–income country, n = 41	6	5	5	4	5	4
Low-income country, n = 30	…	2	3	4	3	3

Normally, vaccines’ COGS provide a floor price for the lowest-income countries and serve as a comparator to the other new vaccines. This is particularly relevant in the lowest-income countries. Considering very preliminary information on vaccine characteristics and manufacturing processes [25], COGS was estimated at $2.50 per dose. It is important to note that at the lowest levels (threshold depending on the vaccine platform in use), the volume of production can have a strong effect on COGS. Consequently, any significant decrease in the anticipated volume of doses sold for an individual manufacturer could result in increased COGS. A higher COGS scenario of $4.00 per dose was also simulated. A lower COGS scenario was not simulated as it would directly result in an improvement of the baseline NPV, assuming no difference in pricing.

As a result of the above considerations, price assumptions by country income group were defined, splitting the countries in 3 groups as represented in Table 3. The analysis assumed the same per-dose price regardless of the number of doses required. Consequently, a vaccine with a 2-dose schedule could enhance the business case, even if that will create additional implementation costs for countries.

**Table 3.** Assumed Price Per Dose of Group B *Streptococcus* Vaccine

Market Segment	Price per Dose
High-income countries, N = 51	$50.00
Upper middle–income countries, N = 22	$15.00
Lower middle–income and low-income countries • Lower middle–income countries (Gavi and non-Gavi eligible), N = 35 • Low-income countries (Gavi eligible), N = 49	$3.50

#### Clinical Development Costs

The total costs and cash outflow for all activities performed as part of the clinical development, for example, clinical trials, regulatory activities, and manufacturing, were estimated as follows: *Phase 1 and 2* completed by 2022 for a total cost of $23.6 million [26] and *phase 3* completed between 2023 and 2027 with 7000 participants and costs of $52.3 million based on the assumption that an immunogenicity trial will be acceptable for primary registration. Marketing authorization based on correlates of protection has been achieved for conjugated vaccines that target encapsulated bacteria similar to GBS. Alternative options to make such a scenario possible for a GBS vaccine have been identified in a recent WHO-sponsored workshop [27]. Nonetheless, this assumption remains relatively uncertain, and a larger and longer efficacy trial may be required. Under a more conservative assumption, 40 000 participants would be needed for a phase 3 trial that will take 8 years over the period 2027–2034 with a total cost of $204.5 million. Those estimates are based on a total cost per participant of $4.612 (The original estimates have been actualized based on the US Bureau of Labor Statistics calculator - https://www.bls.gov/data/inflation_calculator.htm) [28] and include regulatory and manufacturing-related costs.

#### Manufacturing Facility Investment

Between 2023 and 2028, a capital expenditure of $150 million [29, 30] in a high-income setting was estimated to be needed to build the manufacturing plant, procure the necessary equipment, set up and validate the processes, and achieve establishment licensure. The establishment of the manufacturing facility in a country with lower income and costs is also a scenario that will result in lower investments, hence, in the improvement of the overall financial outlook of the initiative.

#### Market Assumptions

The base case scenario was developed under the assumption that only 1 manufacturer would reach the market and commercialize the vaccine for the entire period, hence, benefiting from the total sales of the GBS vaccine. Two more conservative scenarios were simulated based on the base case scenario single-dose assumption: one where a second manufacturer licenses a product in 2034 and, over a 3-year period, gains 40% market share equally split across the 3 market segments (HICs, UMICs, LMICs–LICs) and a second scenario where the first competitor reaches the market in 2031 and a second competitor licenses a product in 2032, reducing the share of the market of the first entrant to 20% by 2033.

#### Other Financial Assumptions

##### Plant Depreciation Rate. 

Using the US Generally Accepted Accounting Practices, a period of 15 years was used to depreciate the facilities.

##### SG&A. 

A proportion of 14% of total revenues was used as the SG&A rate. This percentage is based on the SG&A expenditure reported in 2019 by Sanofi in its annual report for the vaccine business [31].

##### Taxation Rate. 

Using the most recent estimates of the Tax Foundation, the average Organisation for Economic Co-operation and Development taxation rate of 23% was applied [32].

##### Hurdle Rate. 

Based on the WACC of 8.55% calculated by the New York University Stern Business School for pharmaceutical and biotech companies [33], the hurdle rate of 10.5% was used in the calculations consistently with other similar financial evaluation [34].

## RESULTS

The base forecast and scenarios were developed for the period 2029–2040, representing the first decade of vaccine availability. All forecasts were developed under the assumption that the evolution of the global burden of GBS will still warrant a global introduction of the vaccine in 2029 at the completion of the clinical development process.

### Base Demand Results: 1 Dose Delivered During Pregnancy/ANC 1 Dose

Global dose demand in the base case reached more than 40 million doses from 124 countries by 2035 and approximately 110 million doses from 163 countries by 2040. Total demand over the period 2029–2040 was forecast to exceed 560 million doses, 36% from countries in the African region. Current LMICs would account for the largest percentage of demand during the period, forecast as 32% of the total (Figure 2).

### Demand Scenario Results

#### Two Doses Delivered During Pregnancy. 

This scenario resulted in demand for approximately 80 million doses in 2035 and more than 215 million doses in 2040 with a total in excess of 1 billion doses in the period 2029–2040. The lower coverage of the second dose accounted for the forecast being less than 2 times the base forecast.

#### One Dose in Adolescence With HPV Vaccine Plus 1 Dose During Pregnancy. 

Total demand estimates exceeded 80 million doses in 2035 and were in excess of 215 million doses in 2040 with a total in excess of 1 billion doses in the period 2029–2040. Multiple factors influenced this calculation including different target populations and coverage assumptions. The HPV target population was between 4% and 8% higher per year than the ANC target population, while HPV 1 dose coverage was, on average, lower than ANC 1 dose coverage. See the Supplementary Materials for HPV 1 dose coverage data.

### Financial Analysis Results

The NPV of GBS vaccine development and commercialization was calculated for 8 scenarios. The 3 scenarios developed in the demand forecast plus 5 scenarios used to test the impact of the need for an efficacy trial: a higher COGS, the presence of a competitor in the market, the presence of 2 competitors in the market, and the absence of Gavi support.

**Figure 3.**
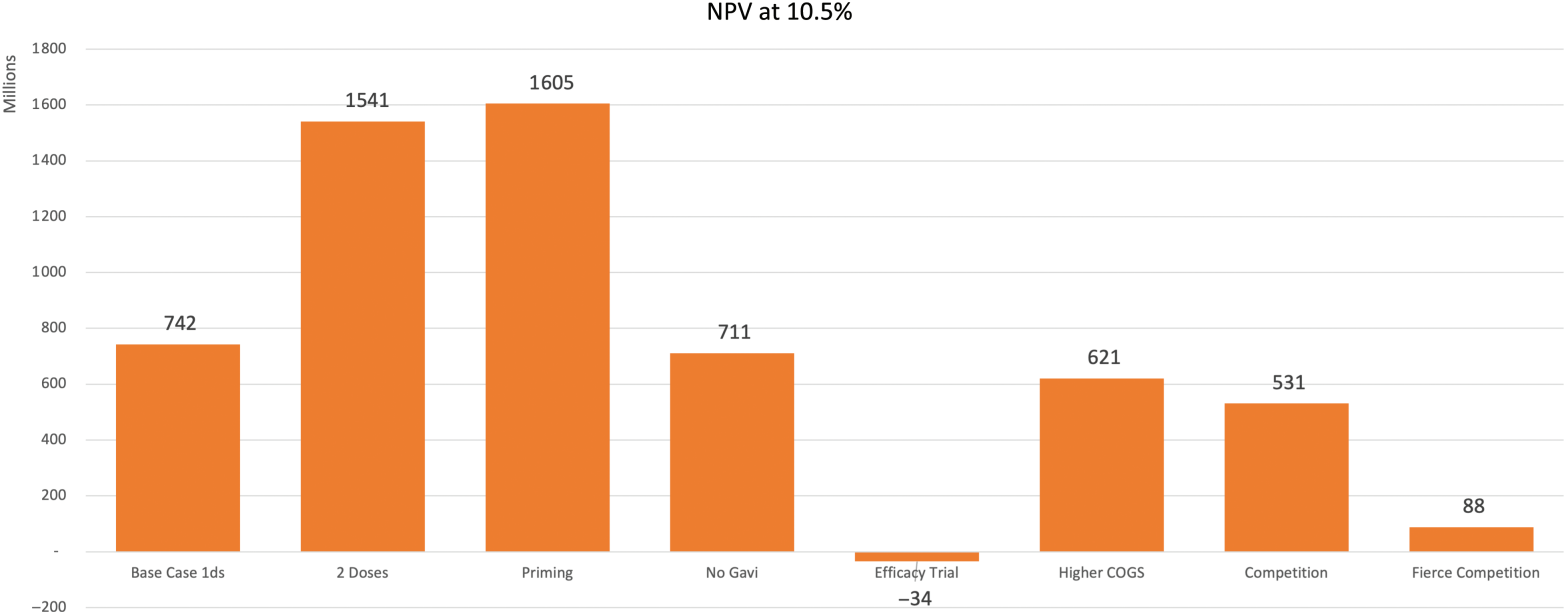
Comparison of NPV and the hurdle rate (10.5%). Abbreviation: 1ds, 1 dose; COGS, cost of goods sold; NPV, net present value.

The NPV was positive in all evaluated scenarios (Figure 3) except for the one where an efficacy trial would be required (The NPV for this scenario is still limited to 2040 with no residual value for consistency with the other scenarios. However, the extended duration of the efficacy trial reduces the number of years where revenues are generated from 12 to 5. With a calculation extended to include 12 years – hence moving beyond 2040 – the NPV remains positive at 285 million USD.) and ranged from $88 million to $1.6 billion, depending on the scenario. These results are highly dependent on revenue from HICs, which represents approximately two-thirds (57%–63%) of all revenues. From a demand standpoint, only a reduction in the total demand to 17% of the base case equally across all income levels led to a negative NPV. On the other hand, the complete absence of HIC demand resulted in the reduction of the NPV by more than 90% to $124 million.

## Discussion

Demand for a GBS vaccine administered during pregnancy and/or in adolescence, assumed to be first licensed in 2028 and introduced at the beginning of 2029, was forecast to exceed 110 million doses by 2040. Demand could reach 220 million doses by 2040 with a 2-dose schedule. This means reaching in excess of 100 million women by 2040. Demand of this size and across countries of all income levels represents an interesting market for vaccine developers.

The strength of this interest is dependent on the level of uncertainty around the most important demand drivers, in order of importance: the number of vaccine doses required per targeted woman, whether and when countries with large populations will introduce the vaccine, and the achievable future (10 to 20 years in the future) coverage for doses administered during ANC visits (ANC dose) and/or in adolescence together with HPV vaccination (HPV dose). The uncertainty around these assumptions also represents the main limitations of the analysis.

For the first parameter, the progress of the necessary clinical trials will provide relevant information to tailor the estimates. For the estimate of the most realistic adoption sequence and of the achievable coverage, further in-depth research will be required. Acceptability studies and use cases analysis could shed light on the size of the target population, while assessment of the cost-effectiveness and perceived value of the vaccine could allow for more precise estimates of the adoption sequence. It is worth noting that the future strength of the maternal platform, in particular, considering the parallel development of an RSV vaccine delivered during pregnancy, can also play an important role in facilitating the adoption of the GBS vaccine.

The financial analysis resulted in a positive NPV for the development of a GBS vaccine across most scenarios, suggesting a high potential for financial viability for this product. Under those scenarios, no additional financial support appears to be required to pursue the development of the GBS vaccine, neither by reducing the cost of clinical development (push funding) nor by de-risking the prospective demand (pull funding).

Also, in this case, those results depend on the following 4 key conditions. (1) *The acceptability of a phase 3 pivotal trial with an immunogenicity end point*. The significantly higher clinical development costs and longer timeline associated with an efficacy trial represent the largest risk to a profitable business model for the first manufacturer (or others that may be in late-stage development prior to licensure of the first vaccine). (2) *Competition to be moderate or absent* in the first 10 years with all global revenue earned by 1 manufacturer or with the first to market retaining a share of 60% or greater. While highly desirable from a supply security standpoint, the presence of a second or third manufacturer in the market would gain market share at the expense of the first and thus lower the overall revenue and financial sustainability for each player. In view of the potential return that can be generated by a GBS vaccine, presence of multiple competitors is to be considered likely. While the first manufacturer’s development costs would remain constant, it may have first-mover advantage in locking certain markets. However, the second or third manufacturer could incur lower development costs, allowing for more aggressive pricing strategies and gain of a relevant share of the market. As result, the presence of 3 competitors in the market would reduce the share of the first to market to 20% by year 5 and its revenues and NPV by three-quarters. It should be noted that while reducing the potential attractiveness of the market, competition and decline in price can speed up the adoption for countries where ability to pay represents a significant barrier. (3) Policy recommendations and use in HICs that provide two-thirds of revenue during the first decade after first registration. Adoption in HICs is necessary to achieve maximum return on investment for the manufacturer. Inability to achieve widespread recommendations or significant lower demand in this market segment because of factors such as vaccine hesitancy and limited support from the medical community would greatly affect the profitability of this vaccine’s business case. (4) Direct margin, based on price and COGS, which are the main drivers of the cash flow generated by the project. A reduction of 60% of prices across all income groups resulted in a NPV loss of $350 million (48% decrease), while a 60% increase in COGS translated to a NPV loss of $120 million (16% decrease).

Since population-based demand forecast and discounted cash flow are standardized and widely accepted modeling approaches for these kinds of analyses, the limitations are primarily linked to the assumptions used in the models. So many years in advance of clinical development completion and vaccine introduction, assumptions can change substantially, including as result of the current coronavirus disease 2019 (COVID-19) pandemic. Assumptions with the largest impact on demand estimates, including the final decisions related to the vaccine schedule and number of doses, country decisions on whether to adopt the vaccine, and the realistic level of achievable coverage, are all based on proxies and the initial knowledge on the vaccine characteristics. Similarly, for the financial variables, the assumptions on competition, price, COGS, and the final clinical trial design are also based on proxies and current knowledge. The progression of clinical development, evolution of the COVID-19 pandemic, and increased country awareness of the program will provide more certainty over time on these assumptions.

## Conclusions

These first estimates of GBS vaccine demand and of the NPV of a vaccine development project suggest the potential for full financial and commercial viability for a vaccine manufacturer to pursue the development and commercialization of a GBS vaccine. This may allow for a business case that is independent of additional financial support. Nonetheless, the role of donors or financers can still prove very important in de-risking the development of the GBS vaccine that, especially at this stage, is still affected by many levels of uncertainty.

More importantly, in view of the relevant level of uncertainty across most of the modeled variables, this analysis points to 5 key risk areas that can affect the GBS vaccine value proposition. These risks include the pivotal phase 3 clinical trial design and costs, the level of competition, the acceptable prices in the various countries, the administration schedule, and the availability of policies at national and global levels that encourage use of the vaccine in high- and low-resource settings, reducing the risk of low uptake, in particular, in high-income settings, critical to ensure a maximum profitability.

To enable equitable access to GBS vaccines, a global, structured policy-making process and a coordinated operational research agenda will be essential for clarifying those areas of uncertainty that are likely to influence the developers’ decisions.

## Supplementary Data

Supplementary materials are available at *Clinical Infectious Diseases* online. Consisting of data provided by the authors to benefit the reader, the posted materials are not copyedited and are the sole responsibility of the authors, so questions or comments should be addressed to the corresponding author.

ciab782_suppl_Supplementary_Materials

## Notes

***Author contributions.*** S.M. and M. M. developed the demand and financial models and performed the analyses to define the assumptions in the second half of 2019 and in the first half of 2020. For the entire duration of the project, C. P. provided input on the demand modeling assumptions. C. M. and P. L. provided guidance on the maternal immunization and group B *Streptococcus* (GBS) policies and impact on demand. All authors discussed the contents of the article and its conclusions and provided feedback and input on the content.

***Acknowledgments.*** We express appreciation to the GBS scientific advisory group members (Martina Lukong Baye, Lumbwe Chola, Kirsty Le Doare, Paul T. Heath, Margaret Ip, Mark Jit, Asma Khalil, Shabir A. Madhi, Liz Mason, Flor Munoz, Maarten Postma, and Stephanie Schrag) for their input and support. Special thanks go to Mark Jiit, Maarten Postma, and Lumbwe Chola for their feedback on the preliminary demand forecast results and to Ranju Baral (PATH) for valuable feedback on the modeling assumptions.

***Supplement sponsorship.*** This supplement is sponsored by Bill & Melinda Gates Foundation.

***Financial support.*** This work was supported by a grant (INV-009018) from the Bill & Melinda Gates Foundation to the WHO and the London School of Hygiene &Tropical Medicine. C. M. reports funding from the WHO. Funding to conduct the survey was received by MMGH Consulting under an Agreement for Performance of Work (ref. Purchase Order nr. 202202102). M. M. reports that this work was supported under a contract of the WHO to MMGH Consulting (WHO work on GBS is financed by a grant from the Bill & Melinda Gates Foundation (INV-007608). MMGH Consulting work is financed by the WHO (via 2 purchase orders, 202309505 and 202482293)

S. M. reports that MMGH Consulting attended and presented to the GBS Scientific Advisory Group financed by the WHO (via purchase order 202309505 mentioned above).

***Potential conflicts of interest.*** P.L. works for the World Health Organization. All remaining authors: No reported conflicts of interest. All authors: No reported conflicts of interest. All authors have submitted the ICMJE Form for Disclosure of Potential Conflicts of Interest. Conflicts that the editors consider relevant to the content of the manuscript have been disclosed.

***Availability of data and material.*** The datasets used and/or analyzed during the current study are available from the corresponding author on reasonable request.

## References

[1] Seale AC , Bianchi-Jassir F, Russell NJ, et al. Estimates of the burden of group B Streptococcal disease worldwide for pregnant women, stillbirths, and children. Clin Infect Dis 2017; 65:200–19. 10.1093/cid/cix664 PMC5849940 29117332

[2] Sinha A , Russell LB, Tomczyk S, et al; GBS Vaccine Cost-Effectiveness Analysis in Sub-Saharan Africa Working Group. Disease burden of group B *Streptococcus* among infants in sub-Saharan Africa: a systematic literature review and meta-analysis. Pediatr Infect Dis J 2016; 35:933–42. 27213263 10.1097/INF.0000000000001233 PMC6858852

[3] Kobayashi M , Schrag SJ, Alderson MR, et al. WHO consultation on group B *Streptococcus* vaccine development: report from a meeting held on 27-28 April 2016. Vaccine 2019; 37:7307–14. 28017431 10.1016/j.vaccine.2016.12.029 PMC6892266

[4] Giersing BK , Modjarrad K, Kaslow DC, Moorthy VS; Committee WHOPDfVA, Committee WHOPDfVPDA. Report from the World Health Organization’s Product Development for Vaccines Advisory Committee (PDVAC) meeting, Geneva, 7-9th September 2015. Vaccine 2016; 34:2865–9. 26993336 10.1016/j.vaccine.2016.02.078 PMC7130468

[5] Vekemans J , Moorthy V, Friede M, et al. Maternal immunization against group B *Streptococcus*: World Health Organization research and development technological roadmap and preferred product characteristics. Vaccine 2019; 37:7391–3. 29398277 10.1016/j.vaccine.2017.09.087 PMC6892248

[6] Lin SM , Zhi Y, Ahn KB, Lim S, Seo HS. Status of group B streptococcal vaccine development. Clin Exp Vaccine Res 2018; 7:76–81. 29399583 10.7774/cevr.2018.7.1.76 PMC5795048

[7] Carreras-Abad C , Ramkhelawon L, Heath PT, Le Doare K. A vaccine against group B *Streptococcus*: recent advances. Infect Drug Resist 2020; 13:1263–72. 32425562 10.2147/IDR.S203454 PMC7196769

[8] Malvolti S , Feiden K. Understanding the vaccine ecosystem: structure and challenges. In: Group S-Avsp, ed. Powering vaccine R&D: opportunities for transformation. Aspen, USA: Aspen Institute, 2021.

[9] United Nations DoEaSA. World Population Prospects 2019; File INT/3-1 Total population by single age, region, subregion and country annually for 1950-2100, Medium variant estimates. Available at: https://www.un.org/en/development/desa/population/publications/database/index.asp.

[10] Zuber PL , Dumolard L, Shirey M, Rizzo I, Marshall J. Forecasting demand for Hib-containing vaccine in the world’s poorest countries: a 4-year prospective experience. Vaccine 2009; 27:410–5. 19013493 10.1016/j.vaccine.2008.10.069

[11] Amarasinghe A , Wichmann O, Margolis HS, Mahoney RT. Forecasting dengue vaccine demand in disease endemic and non-endemic countries. Hum Vaccin 2010; 6:745–53. 10.4161/hv.6.9.12587 PMC3056060 20930501

[12] Cernuschi T , Malvolti S, Nickels E, Friede M. Bacillus Calmette-Guérin (BCG) vaccine: a global assessment of demand and supply balance. Vaccine 2018; 36:498–506. 29254839 10.1016/j.vaccine.2017.12.010 PMC5777639

[13] Gotze U , Northcott D, Schuster P Discounted cash flow methods. Investment appraisal. Germany: Springer International Publishing AG, 2015:47–83.

[14] World Health Organization. WHO preferred product characteristics for group B *Streptococcus* vaccines. Available at: https://apps.who.int/iris/handle/10665/258703. Accessed November 2020.

[15] Lawn JE , Blencowe H, Waiswa P, et al Stillbirths: rates, risk factors, and acceleration towards 2030. Lancet 2016; 387:587–603. 26794078 10.1016/S0140-6736(15)00837-5

[16] National Center for Health Statistics. Multiple births in the United States. Available at: https://www.cdc.gov/nchs/fastats/multiple.htm. Accessed November 2020.

[17] World Health Organization. Global Market Study HPV. Available at: https://www.who.int/immunization/programmes_systems/procurement/mi4a/platform/module2/HPV_Global_Market_Study_Public_Summary-Nov2020.pdf?ua=1. Accessed November 2020.

[18] Baral R , Fleming J, Khan S, Higgins D, Hendrix N, Pecenka C. Inferring antenatal care visit timing in low- and middle-income countries: methods to inform potential maternal vaccine coverage. PLoS One 2020; 15:e0237718. 32817688 10.1371/journal.pone.0237718 PMC7446781

[19] UNICEF. Maternal and newborn health coverage database. Available at: https://data.unicef.org/topic/maternal-health/antenatal-care/. Accessed 2019.

[20] Jowett M , Brunal M, Flores G, Cylus J. Spending targets for health: no magic number. Health financing working paper No 1. World Health Organization, 2016.

[21] World Bank. Domestic general government health expenditure (% of general government expenditure ). Available at: https://data.worldbank.org/indicator/SH.XPD.GHED.GE.ZS. Accessed November 2020.

[22] World Health Organization. Maternal immunization and antenatal care situation analysis: report of the MIACSA project, 2016–2019. 2020. Available at: https://www.who.int/publications/i/item/9789240004016. Accessed November 2020.

[23] Market Information for Access to Vaccines (MI4A). Global HPV vaccine market study— demand forecasting methodology. In: World Health Organization, ed. SAGE Meeting, October 2019, 2019. Available at: https://www.who.int/immunization/sage/meetings/2019/october/7_Global_HPV_Vaccine_Market_Study_-_Demand_Methodology.pdf.

[24] World Health Organization. Revising global indicative wastage rates: a WHO initiative for better planning and forecasting of vaccine supply needs. 2019. Available at: https://www.who.int/immunization/programmes_systems/supply_chain/resources/Revising_Wastage_Concept_Note.pdf. Accessed November 2020.

[25] Absalon J , Segall N, Block SL, et al. Safety and immunogenicity of a novel hexavalent group B *Streptococcus* conjugate vaccine in healthy, non-pregnant adults: a phase 1/2, randomised, placebo-controlled, observer-blinded, dose-escalation trial. Lancet Infect Dis 2021; 21:263–74. 32891191 10.1016/S1473-3099(20)30478-3 PMC9760110

[26] Gouglas D , Thanh Le T, Henderson K, et al. Estimating the cost of vaccine development against epidemic infectious diseases: a cost minimisation study. Lancet Glob Health 2018; 6:e1386–96. 30342925 10.1016/S2214-109X(18)30346-2 PMC7164811

[27] Vekemans J , Crofts J, Baker CJ, et al. The role of immune correlates of protection on the pathway to licensure, policy decision and use of group B *Streptococcus* vaccines for maternal immunization: considerations from World Health Organization consultations. Vaccine 2019; 37:3190–8. 31031031 10.1016/j.vaccine.2019.04.039 PMC6528168

[28] Light DW , Andrus JK, Warburton RN. Estimated research and development costs of rotavirus vaccines. Vaccine 2009; 27:6627–33. 19665605 10.1016/j.vaccine.2009.07.077

[29] Plotkin S , Robinson JM, Cunningham G, Iqbal R, Larsen S. The complexity and cost of vaccine manufacturing— an overview. Vaccine 2017; 35:4064–71. 10.1016/j.vaccine.2017.06.003 PMC5518734 28647170

[30] Wilson P . Giving developing countries the best shot: an overview of vaccine access and R&D: campaign for access to essential Medicines Médecins Sans Frontières. 2010. Available at: https://msfaccess.org/giving-developing-countries-best-shot-overview-vaccine-access-and-rd. Accessed November 2020

[31] Sanofi. Form 20-F - 2019 annual report. 2020. Available at: https://www.sanofi.com/-/media/Project/One-Sanofi-Web/Websites/Global/Sanofi-COM/Home/common/docs/investors/2020_03_23_Sanofi-Report-2019-20F-accessible.pdf?la=en&hash=14ACCB31D1FFFF966C8EA96CA7EE5049. Accessed May 2021.

[32] Asen E . Corporate tax rates around the world, 2019. 2019. Available at: https://taxfoundation.org/corporate-tax-rates-around-the-world-2019/. Accessed May 2021.

[33] Damodaran A . Cost of capital by sector (US). Available at: http://people.stern.nyu.edu/adamodar/New_Home_Page/datafile/wacc.htm. Accessed May 2021.

[34] DiMasi JA , Grabowski HG, Hansen RW. Innovation in the pharmaceutical industry: new estimates of R&D costs. J Health Econ 2016; 47:20–33. 26928437 10.1016/j.jhealeco.2016.01.012

