# Supplementary material for: A Financial and Global Demand Analysis to Inform Decisions for Funding and Clinical Development of Group B Streptococcus Vaccines for Pregnant Women
Source: Clin Infect Dis. 2021 Nov 2;74(Suppl 1):S70–9. doi: 10.1093/cid/ciab782 (PMC8775646; doi:10.1093/cid/ciab782)
Supplement: ciab782_suppl_Supplementary_Materials [file ciab782_suppl_supplementary_materials.docx]

**SUPPLEMENT TITLE:** Group B Streptococcal disease for Pregnant Women and Children Worldwide

**PAPER TITLE:** A financial and global demand analysis to inform decisions for funding and clinical development of GBS vaccines for pregnant women

**AUTHORS:** Stefano Malvolti^1^, Clint Pecenka^2^, Carsten Mantel^1^, Melissa Malhame^1^, Philipp Lambach^3^

**Affiliations:**

1. MMGH Consulting, Zurich, Switzerland
2. PATH, Seattle, USA
3. Department of Immunization, Vaccines and Biologicals, World Health Organization, Geneva, Switzerland

**Corresponding Author** Stefano Malvolti, Managing Director & Cofounder, MMGH Consulting, Kubergstrasse 1, 8049 Zurich, Switzerland, [malvoltis@mmglobalhealth.org](mailto:malvoltis@mmglobalhealth.org), +41 43 8345678

**Keywords**: Group B Strep, neurodevelopment, neonatal sepsis, forecast, demand, financial evaluation, vaccine

**Running Title:** Financial and global demand analysis iGBS

Table of Contents

[Supplementary Annex Table 1: Estimated coverage rate per country 3](#_Toc78957827)

# Supplementary Annex Table 1: Estimated coverage rate per country

| **ISO** | **Countries and areas** | **Year** | **Long Source** | **ANC4** |
| --- | --- | --- | --- | --- |
| AFG | Afghanistan | 2015 | Demographic and Health Survey 2015 | 18% |
| AGO | Angola | 2015 | Demographic and Health Survey 2015 | 61% |
| ALB | Albania | 2018 | Demographic and Health Survey 2017-2018 | 78% |
| ARG | Argentina | 2012 | Multiple Indicator Cluster Survey 2011-2012 | 90% |
| ARM | Armenia | 2016 | Demographic and Health Survey 2015-2016 | 96% |
| ATG | Antigua and Barbuda | 2013 | WHO Health Situation in the Americas Basic Indicators 2014 | 100% |
| AUS | Australia | 2008 | Other NS 2008 | 92% |
| AZE | Azerbaijan | 2011 | Demographic and Health Survey 2011 | 66% |
| BDI | Burundi | 2016 | Demographic and Health Survey 2016 | 49% |
| BEN | Benin | 2018 | Demographic and Health Survey 2017-2018 | 52% |
| BFA | Burkina Faso | 2015 | Demographic and Health Survey (Preliminary) 2015 | 47% |
| BGD | Bangladesh | 2014 | Demographic and Health Survey 2014 | 31% |
| BGR | Bulgaria | 2018 | WHO/UNICEF est | 92% |
| BHR | Bahrain | 2013 | Health Summary Statistics 2013 | 100% |
| BHS | Bahamas | 2013 | WHO Health Situation in the Americas Basic Indicators 2014 | 85% |
| BIH | Bosnia and Herzegovina | 2012 | Multiple Indicator Cluster Survey 2011-2012 | 84% |
| BLR | Belarus | 2012 | Multiple Indicator Cluster Survey 2012 | 100% |
| BLZ | Belize | 2016 | Multiple Indicator Cluster Survey 2015-2016 | 93% |
| BOL | Bolivia (Plurinational State of) | 2016 | Encuesta de Demografia y Salud (EDSA ) 2016 | 86% |
| BRA | Brazil | 2015 | MoH SIMSISC (Prelim) 2014-2015 | 91% |
| BRB | Barbados | 2012 | Multiple Indicator Cluster Survey 2012 | 88% |
| BRN | Brunei Darussalam | 2012 | HIB 2012 | 93% |
| BTN | Bhutan | 2015 | National Nutrition Survey 2015 | 85% |
| BWA | Botswana | 2007 | Family Health Survey 2007 | 73% |
| CAF | Central African Republic | 2010 | Multiple Indicator Cluster Survey 2010 | 38% |
| CAN | Canada | 2007 | Other NS 2006-2007 | 99% |
| CHN | China | 2013 | National Health Commission, National Health Services Survey (NHSS) 2013 | 81% |
| CIV | Côte d'Ivoire | 2016 | Multiple Indicator Cluster Survey 2016 | 51% |
| CMR | Cameroon | 2014 | Multiple Indicator Cluster Survey 2014 | 59% |
| COD | Democratic Republic of the Congo | 2014 | Demographic and Health Survey 2013-2014 | 48% |
| COG | Congo | 2015 | Multiple Indicator Cluster Survey 2014-2015 | 79% |
| COL | Colombia | 2016 | Demographic and Health Survey 2015-2016 | 90% |
| COM | Comoros | 2012 | Demographic and Health Survey, Multiple Indicator Cluster Survey 2012 | 49% |
| CRI | Costa Rica | 2011 | Multiple Indicator Cluster Survey 2011 | 90% |
| CUB | Cuba | 2014 | Multiple Indicator Cluster Survey 2014 | 98% |
| CZE | Czechia | 1993 | Other NS 1993 | 97% |
| DEU | Germany | 2014 | QUAG 2014 | 99% |
| DJI | Djibouti | 2012 | EDSFPAPFAM (Prelim) 2012- 2012 | 23% |
| DOM | Dominican Republic | 2014 | Multiple Indicator Cluster Survey 2014 | 93% |
| DZA | Algeria | 2012 | Multiple Indicator Cluster Survey 2012 | 67% |
| ECU | Ecuador | 2004 | Other NS 2004 | 58% |
| EGY | Egypt | 2014 | Demographic and Health Survey 2014 | 83% |
| ERI | Eritrea | 2010 | n Population and Health Survey 2010 | 57% |
| EST | Estonia | 2014 | Medical Birth Registry 2014 | 97% |
| ETH | Ethiopia | 2016 | Demographic and Health Survey 2016 | 32% |
| FJI | Fiji | 2013 | National Immunisation Coverage Survey 2013 | 94% |
| FRA | France | 2010 | Enquête nationale périnatale 2010 | 99% |
| FSM | Micronesia | 2018 | WHO/UNICEF est | 75% |
| GAB | Gabon | 2012 | Demographic and Health Survey 2012 | 78% |
| GEO | Georgia | 2016 | HSCSY 2015 | 88% |
| GHA | Ghana | 2014 | Demographic and Health Survey 2014 | 87% |
| GIN | Guinea | 2018 | Demographic and Health Survey 2018 | 35% |
| GMB | Gambia | 2013 | Demographic and Health Survey 2013 | 78% |
| GNB | Guinea-Bissau | 2014 | Multiple Indicator Cluster Survey 2014 | 65% |
| GNQ | Equatorial Guinea | 2011 | Demographic and Health Survey 2011 | 67% |
| GTM | Guatemala | 2015 | Demographic and Health Survey 2014-2015 | 86% |
| GUY | Guyana | 2014 | Multiple Indicator Cluster Survey 2014 | 87% |
| HND | Honduras | 2012 | Demographic and Health Survey 2011-2012 | 89% |
| HRV | Croatia | 2018 | n Institute for Public Health 2018 | 98% |
| HTI | Haiti | 2017 | Demographic and Health Survey 2016-2017 | 67% |
| IDN | Indonesia | 2017 | Demographic and Health Survey 2017 | 77% |
| IND | India | 2016 | National Family Health Survey (Demographic and Health Survey) 2015-2016 | 51% |
| IRN | Iran (Islamic Republic of) | 2005 | Ministry of Health 2005 | 94% |
| IRQ | Iraq | 2018 | Multiple Indicator Cluster Survey 2018 | 68% |
| ITA | Italy | 2003 | Ministry of Health 2003 | 68% |
| JAM | Jamaica | 2011 | Multiple Indicator Cluster Survey 2011 | 86% |
| JOR | Jordan | 2017 | Demographic and Health Survey 2017 | 92% |
| KAZ | Kazakhstan | 2015 | Multiple Indicator Cluster Survey 2015 | 95% |
| KEN | Kenya | 2014 | Demographic and Health Survey 2014 | 58% |
| KGZ | Kyrgyzstan | 2014 | Multiple Indicator Cluster Survey 2014 | 95% |
| KHM | Cambodia | 2014 | Demographic and Health Survey 2014 | 76% |
| KIR | Kiribati | 2009 | Demographic and Health Survey 2009 | 71% |
| KOR | Republic of Korea | 2012 | National Health Survey 2012 | 97% |
| LAO | Lao People's Democratic Republic | 2017 | Multiple Indicator Cluster Survey 2017 | 62% |
| LBN | Lebanon | 2018 | WHO/UNICEF est | 83% |
| LBR | Liberia | 2013 | Demographic and Health Survey 2013 | 78% |
| LBY | Libya | 2018 | WHO/UNICEF est | 97% |
| LCA | Saint Lucia | 2012 | Multiple Indicator Cluster Survey 2012 | 90% |
| LKA | Sri Lanka | 2007 | Demographic and Health Survey 2006-2007 | 93% |
| LSO | Lesotho | 2014 | Demographic and Health Survey 2014 | 74% |
| LUX | Luxembourg | 2011 | National Health Survey 2011 | 97% |
| MAR | Morocco | 2018 | Enquête Nationale surla Population et la Santé Familiale 2018 | 54% |
| MDA | Republic of Moldova | 2012 | Multiple Indicator Cluster Survey 2012 | 95% |
| MDG | Madagascar | 2013 | L’Enque^te Nationale sur le Suivi des indicateurs des Objectifs du Mille´naire pour le De´veloppement 2012-2013 | 51% |
| MDV | Maldives | 2017 | Demographic and Health Survey 2016-2017 | 82% |
| MEX | Mexico | 2015 | Multiple Indicator Cluster Survey 2015 | 94% |
| MHL | Marshall Islands | 2007 | Demographic and Health Survey 2007 | 77% |
| MKD | Republic of North Macedonia | 2011 | Multiple Indicator Cluster Survey 2011 | 94% |
| MLI | Mali | 2018 | Demographic and Health Survey 2018 | 43% |
| MMR | Myanmar | 2018 |  | 90% |
| MNE | Montenegro | 2013 | Multiple Indicator Cluster Survey 2013 | 87% |
| MNG | Mongolia | 2013 | Multiple Indicator Cluster Survey 2013 | 90% |
| MOZ | Mozambique | 2015 | AIDS Indicator Survey 2015 | 52% |
| MRT | Mauritania | 2015 | Multiple Indicator Cluster Survey 2015 | 63% |
| MUS | Mauritius | 2018 |  | 95% |
| MWI | Malawi | 2016 | Demographic and Health Survey 2015-2016 | 51% |
| NAM | Namibia | 2013 | Demographic and Health Survey 2013 | 63% |
| NER | Niger | 2015 | Etude Nationale d’Evaluation d’Indicateurs SocioEconomiques et Démographiques 2015 | 38% |
| NGA | Nigeria | 2018 | Demographic and Health Survey 2018 | 57% |
| NIC | Nicaragua | 2012 | ENDESA (Prelim) 2011-2012 | 88% |
| NPL | Nepal | 2017 | Demographic and Health Survey 2016-2017 | 69% |
| NRU | Nauru | 2007 | Demographic and Health Survey 2007 | 40% |
| OMN | Oman | 2014 | Multiple Indicator Cluster Survey 2014 | 94% |
| PAK | Pakistan | 2018 | Demographic and Health Survey 2017-2018 | 51% |
| PAN | Panama | 2013 | Multiple Indicator Cluster Survey 2013 | 88% |
| PER | Peru | 2014 | Demographic and Health Survey 2016 | 96% |
| PHL | Philippines | 2017 | Demographic and Health Survey 2017 | 87% |
| PLW | Palau | 2010 | WHO Western Pacific Country Health Information Profile 2011 | 81% |
| PNG | Papua New Guinea | 2018 | Demographic and Health Survey 2016-2018 | 49% |
| PRK | Democratic People's Republic of Korea | 2017 | Multiple Indicator Cluster Survey 2017 | 94% |
| PRY | Paraguay | 2016 | Multiple Indicator Cluster Survey 2016 | 94% |
| QAT | Qatar | 2012 | Multiple Indicator Cluster Survey 2012 | 85% |
| RKS | Kosovo | 2014 | Multiple Indicator Cluster Survey 2013-2014 | 92% |
| ROU | Romania | 2004 | Other NS 2004 | 76% |
| RUS | Russia | 2018 | WHO/UNICEF est | 97% |
| RWA | Rwanda | 2015 | Demographic and Health Survey 2014-2015 | 44% |
| SDN | Sudan | 2014 | Multiple Indicator Cluster Survey 2014 | 51% |
| SEN | Senegal | 2017 | Demographic and Health Survey 2017 | 57% |
| SLB | Solomon Islands | 2015 | Demographic and Health Survey 2015 | 69% |
| SLE | Sierra Leone | 2017 | Multiple Indicator Cluster Survey 2017 | 78% |
| SLV | El Salvador | 2014 | Multiple Indicator Cluster Survey 2014 | 90% |
| SOM | Somalia | 2006 | Multiple Indicator Cluster Survey 2006 | 6% |
| SRB | Serbia | 2014 | Multiple Indicator Cluster Survey 2014 | 94% |
| SSD | South Sudan | 2010 | Multiple Indicator Cluster Survey 2010 | 17% |
| STP | Sao Tome and Principe | 2014 | Multiple Indicator Cluster Survey 2014 | 84% |
| SUR | Suriname | 2010 | Multiple Indicator Cluster Survey 2010 | 67% |
| SWZ | Eswatini | 2014 | Multiple Indicator Cluster Survey 2014 | 76% |
| SYR | Syrian Arab Republic | 2009 | Household and Health Survey 2010 | 64% |
| TCD | Chad | 2015 | Demographic and Health Survey 2014-2015 | 31% |
| TGO | Togo | 2014 | Demographic and Health Survey 2013-2014 | 57% |
| THA | Thailand | 2016 | Multiple Indicator Cluster Survey 2015-2016 | 91% |
| TJK | Tajikistan | 2017 | Demographic and Health Survey 2017 | 64% |
| TKM | Turkmenistan | 2016 | Multiple Indicator Cluster Survey 2015-2016 | 96% |
| TLS | Timor-Leste | 2016 | Demographic and Health Survey 2016 | 77% |
| TON | Tonga | 2012 | Demographic and Health Survey 2012 | 70% |
| TTO | Trinidad and Tobago | 2013 | WHO Health Situation in the Americas Basic Indicators 2014 | 100% |
| TUN | Tunisia | 2012 | Multiple Indicator Cluster Survey 2011-2012 | 85% |
| TUR | Turkey | 2014 | Demographic and Health Survey 2013-2014 | 89% |
| TUV | Tuvalu | 2007 | Demographic and Health Survey 2007 | 67% |
| TZA | United Republic of Tanzania | 2017 | Malaria Indicator Survey 2017 | 62% |
| UGA | Uganda | 2016 | Demographic and Health Survey 2016 | 60% |
| UKR | Ukraine | 2012 | Multiple Indicator Cluster Survey 2012 | 87% |
| URY | Uruguay | 2013 | Multiple Indicator Cluster Survey 2012-2013 | 77% |
| USA | United States | 2011 | WHO Health Situation in the Americas Basic Indicators 2014 | 97% |
| UZB | Uzbekistan | 1996 | Demographic and Health Survey 1996 | 79% |
| VCT | Saint Vincent and the Grenadines | 2009 | WHO Health Situation in the Americas Basic Indicators 2010 | 100% |
| VEN | Venezuela (Bolivarian Republic of) | 2013 | MPPS Comunicación 0625 2017 | 84% |
| VNM | Viet Nam | 2014 | Multiple Indicator Cluster Survey 2013-2014 | 74% |
| VUT | Vanuatu | 2013 | Demographic and Health Survey 2013 | 52% |
| WSM | Samoa | 2014 | Demographic and Health Survey 2014 | 73% |
| YEM | Yemen | 2013 | Demographic and Health Survey 2013 | 25% |
| ZAF | South Africa | 2016 | Demographic and Health Survey 2016 | 76% |
| ZMB | Zambia | 2014 | Demographic and Health Survey 2013-2014 | 56% |
| ZWE | Zimbabwe | 2015 | Demographic and Health Survey 2015 | 76% |
